# Human-Induced Pluripotent Stem Cell-Derived Neural Organoids as a Novel In Vitro Platform for Developmental Neurotoxicity Assessment

**DOI:** 10.3390/ijms252312523

**Published:** 2024-11-21

**Authors:** Tsunehiko Hongen, Kenta Sakai, Tomohiro Ito, Xian-Yang Qin, Hideko Sone

**Affiliations:** 1 Environmental Health and Prevention Research Unit, Yokohama University of Pharmacy, 601 Matano, Totsuka, Yokohama 245-0066, Japan; 220107_mw@yok.hamayaku.ac.jp (T.H.); kenta.sakai@yok.hamayaku.ac.jp (K.S.); 2 Center for Health and Environmental Risk Research, National Institute for Environmental Studies, 16-2 Onogawa, Tsukuba 305-8506, Japan; itotomo@nies.go.jp; 3 Laboratory for Cellular Function Conversion Technology, RIKEN Center for Integrative Medical Sciences, 1-7-22 Suehiro, Tsurumi, Yokohama 230-0045, Japan; xyqin@riken.jp

**Keywords:** human induced pluripotent stem cells, neural organoid, in vitro, developmental neurotoxicity

## Abstract

There has been a recent drive to replace in vivo studies with in vitro studies in the field of toxicity testing. Therefore, instead of conventional animal or planar cell culture models, there is an urgent need for in vitro systems whose conditions can be strictly controlled, including cell–cell interactions and sensitivity to low doses of chemicals. Neural organoids generated from human-induced pluripotent stem cells (iPSCs) are a promising in vitro platform for modeling human brain development. In this study, we developed a new tool based on various iPSCs to study and predict chemical-induced toxicity in humans. The model displayed several neurodevelopmental features and showed good reproducibility, comparable to that of previously published models. The results revealed that basic fibroblast growth factor plays a key role in the formation of the embryoid body, as well as complex neural networks and higher-order structures such as layered stacking. Using organoid models, pesticide toxicities were assessed. Cells treated with low concentrations of rotenone underwent apoptosis to a greater extent than those treated with high concentrations of rotenone. Morphological changes associated with the development of neural progenitor cells were observed after exposure to low doses of chlorpyrifos. These findings suggest that the neuronal organoids developed in this study mimic the developmental processes occurring in the brain and nerves and are a useful tool for evaluating drug efficacy, safety, and toxicity.

## 1. Introduction

Currently, there are limited effective treatments or preventive strategies for neurological disorders caused by exposure to environmental chemicals, such as endocrine disruptors or pesticides [1,2,3]. The complexity of the brain makes it challenging to develop targeted interventions; moreover, while certain treatments show promise in animal models, their efficacy often does not translate to humans [4]. Therefore, three-dimensional neural organoid models, which are brain-like tissues generated from induced pluripotent stem cells (iPSCs) that do not require the use of animal sources, have attracted attention as a potential means to enhance our understanding of brain function, neurogenesis, and chemical-induced toxicity prediction [5,6]. This is regarded as an advanced in vitro method based on novel human cells. The key feature of organoids is the autonomous generation of tissue bodies that mimic the spontaneous formation of bodies and organs in accordance with the cell’s own program. This feature may facilitate the evaluation of the effects or side effects of new chemical substances under conditions that are more representative of the in vivo environment by accurately mimicking the responses of actual tissues [7]. Thus, these models are expected to improve researchers’ ability to select promising drug development candidates and simultaneously predict potential toxicities. This will facilitate rapid and safe drug development, and these models are therefore expected to make a significant contribution to the advancement of research and therapeutics in the medical field.

The developing brain has the capacity to generate new neurons and form and strengthen existing neural circuits. Neural circuits are continually altered via learning and experience, and appropriate stimulation and experience can significantly impact brain development [8]. In contrast, in the adult brain, the generation of new neurons is limited, and the main focus is on changing existing neural circuits and repairing synapses [9,10]. In addition, the developing brain, which is less mature, is considered more vulnerable to external influences [11]. The effects of neurotoxic substances and various environmental stresses on the developing brain are likely to be more severe and long-lasting than those on the adult brain. Harmful environmental conditions, poor nutrition, and chemical exposure can disturb the formation of neural circuits and functions in the developing brain [12]. Thus, unknown developmental neurotoxicity and potential neurotoxic effects of certain chemicals and drugs pose a major concern.

There are several issues with the current developmental neurotoxicity testing (DNT) guidelines [13]. DNT is primarily based on rodent studies; however, as the nervous systems of animals and humans are distinct, the results of animal studies do not always translate directly to humans [14]. Specifically, the human nervous system is complex and diverse, which makes it difficult to encompass all aspects within a single animal model. For example, human brains are larger, more complex, and take longer to develop; conversely, rodent brains are relatively small and develop more rapidly. Moreover, there exist differences in the type and arrangement of neurotransmitters and receptors, which may be crucial factors for assessing the effects of drugs and chemicals [15,16].

The objective of this paper is to enhance DNT assessment methods by leveraging human-derived neural organoids as a model to evaluate the effects of pesticides on the developing brain. Traditional DNT assessments are limited, as they typically capture only a snapshot of toxicity effects at a single point in time, without addressing the dynamic, ongoing changes in the developing brain [17,18]. This limitation makes it challenging to accurately replicate the stages of brain development and capture the nuanced effects that may occur during different growth phases. Additionally, the absence of reliable methods and guidelines for assessing the effects of chemical mixtures further highlights the gap between laboratory testing and realistic environmental conditions [19].

To address these challenges, this study developed human iPSC-derived neural organoid models that better mimic the structural and functional properties of the human brain during early development. These organoids serve as a more accurate and responsive model for assessing toxicity and evaluating drug efficacy and safety, as well as potential neurotoxic effects of environmental chemicals, specifically pesticides. By providing a three-dimensional, human-relevant platform, these neural organoids offer a valuable tool for understanding the impacts of various substances on neurodevelopment, which may ultimately contribute to safer chemical regulations and improved public health outcomes.

## 2. Results

### 2.1. Effects of Growth Factors on the Surface Construction of Neural Organoids

**Figure 1:**
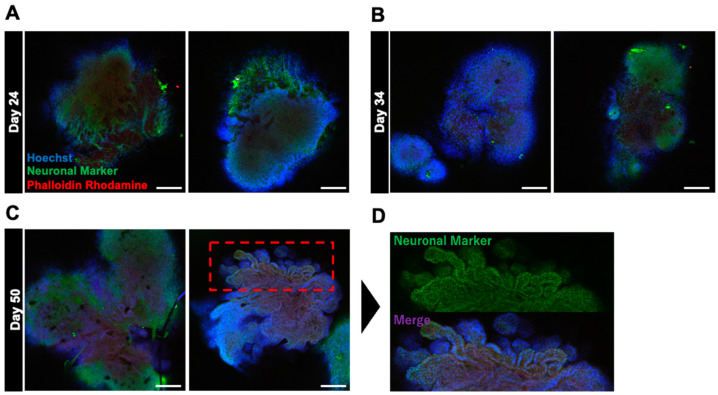
Immunostaining images depicting the effect of bFGF on organoid differentiation at various stages. (**A**–**C**) present immunostained images of organoids on days 24, 34, and 50, respectively. (**D**) presents a magnified view of the upper part of the bFGF-exposed organoid on day 50. Staining included Hoechst staining (in blue) for nuclear labeling, neuron markers (in green) for neuron identification, and phalloidin–rhodamine (in red) for cytoskeletal visualization. Images were captured using Olympus FV10-ASW (10× objective; Scale bar, 200 μm).

Basic fibroblast growth factor (bFGF) has a specific effect on the superficial construction of neural organoids [8,20]. Therefore, we hypothesized that bFGF exposure can promote the formation of cortical layers and accelerate the layered arrangement and structural maturation of neurons. To test our hypothesis, we performed a detailed analysis of changes in the surface layer architecture of neural organoids grown in culture medium supplemented with bFGF (Figure 1). bFGF at a concentration of 4 ng/mL was added to the culture medium from days 0 to 4 of the embryoid body (EB) formation phase. bFGF formed distinct edges and three-dimensional structures, indicating that EBs were growing normally. The internal structure was analyzed using fluorescence immunostaining, and neurites were observed inside the cells on day 24 of differentiation. To achieve glial cell differentiation, neuronal development, and structural maturation, the cells were cultured for up to 50 days. On day 50, the cells formed a novel folded structure, displaying a wrinkled appearance similar to that of the human brain.

### 2.2. Fabrication and Analysis of the Internal Structure of Neural Organoids

**Figure 2:**
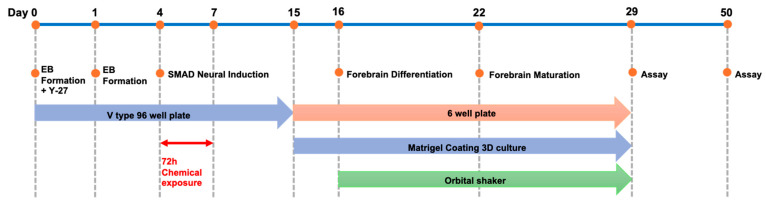
Forebrain organoid differentiation protocol. Cells were seeded at a density of 9000 cells per well, and embryoid bodies were allowed to form until day 4 of culture. The cells were then naturally induced in SMAD (Sma and Mad related protein) inhibitor-containing medium until day 15, differentiated into forebrain-type organoids from day 16, and cultured to maturity from days 22 to 29. Maintenance culture was continued until day 50.

The neurospheroids developed in previous studies lack multicellular interactions and a physiological environment. Therefore, to create a model of complex neural tissue structure that permits safety evaluations, we fabricated neural organoids with mixed glial cells and other cells (Figure 2).

**Figure 3:**
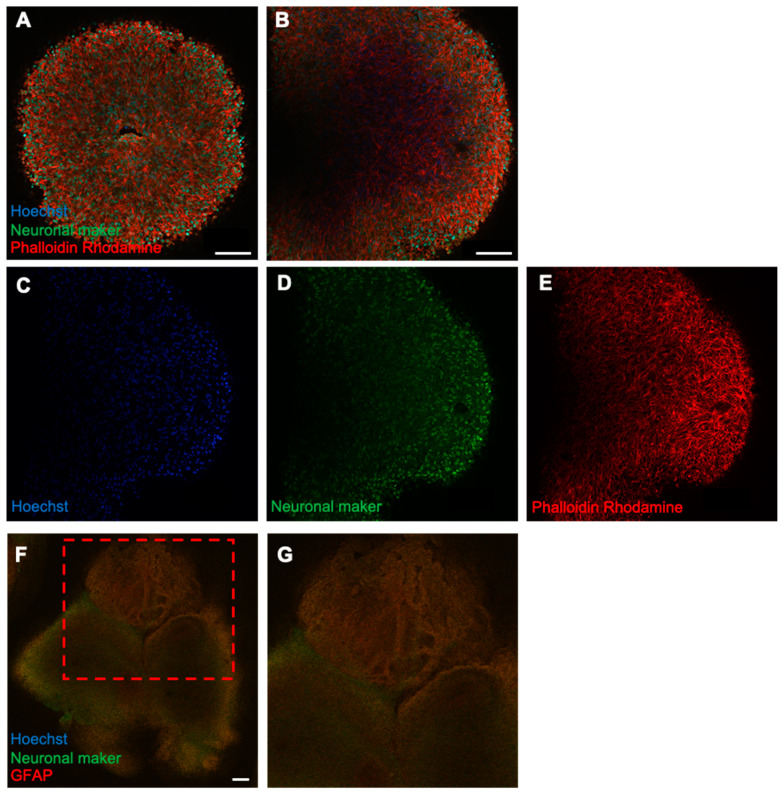
Fluorescence immunostaining of forebrain-type organoids on day 50 of culture. (**A**) Internal structure of the organoids on day 50 of culture, as observed using a confocal microscope Z-stack with a 25× objective. (**B**) Magnified image using a 40× objective. (**C**–**E**) Localization of cells by each marker. Cell nuclei (blue), neurons (green), and cytoskeleton (red) are shown (4× objective; Scale bar, 100 μm. (**F**) A forebrain organoid stained with the glial cell marker GFAP (red) and neurons (green). (**F**,**G**) An enlarged view of the upper part (4× objective; Scale bar, 200 μm).

To examine neuronal development and cellular composition, fluorescence immunostaining of neural organoids was performed. On day 50 of culture, strong staining of the cytoskeletal marker phalloidin–rhodamine (red) was observed throughout the organoids (Figure 3). This suggested that microtubules and microfilaments were developing in the organoids. Moreover, scattered neuronal markers (beta-tubulin III, green) were observed, and the positively stained cells lacked dendrites, which are characteristic of well-developed neurons, indicating that the cells were not functionally mature.

### 2.3. Rotenone Suppresses Neurodevelopment and the Expression of Synaptic Transmission-Related Genes

**Figure 4:**
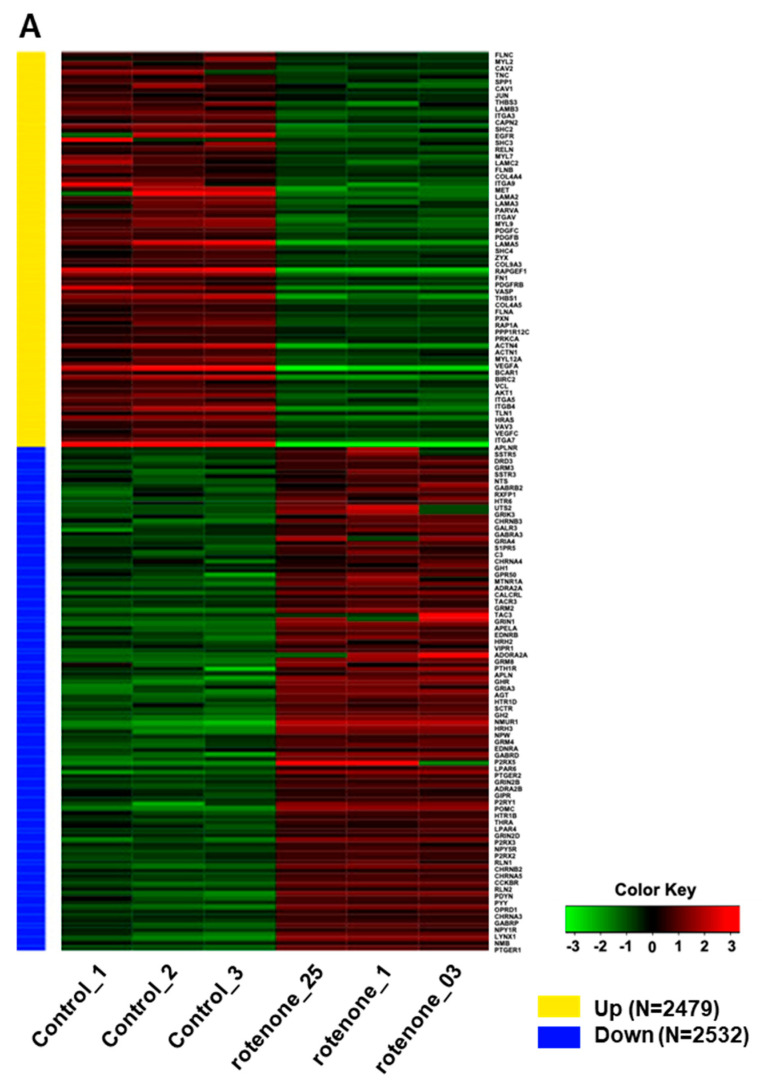

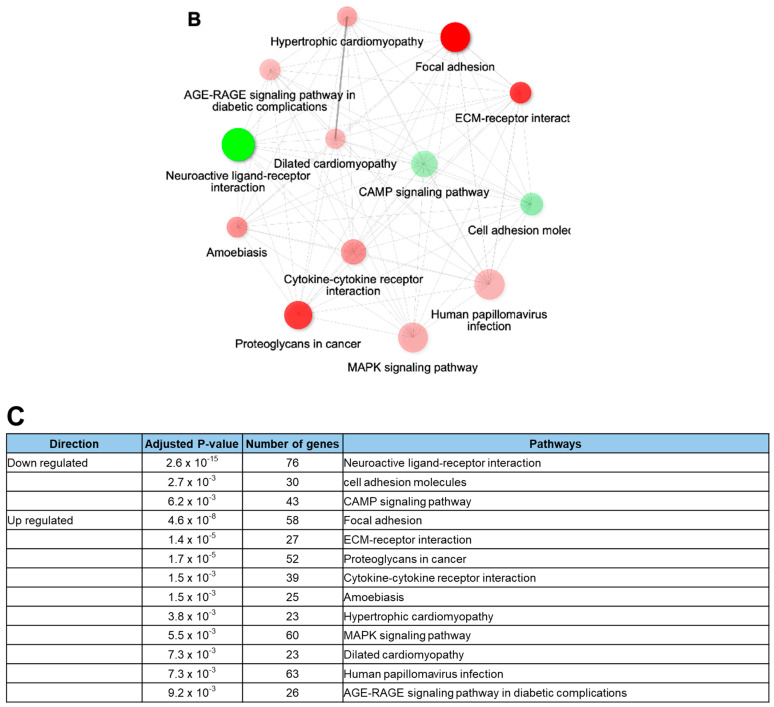
RNA sequencing analysis of human-induced pluripotent stem cells exposed to rotenone. (**A**) Heatmap illustrating the differential gene expression between control and rotenone-treated samples. Genes are ranked based on their standard deviation across all samples, and the top 1000 genes are used for hierarchical clustering analysis. Details of which genes are upregulated or downregulated in the control versus rotenone treatment are provided in the table within the Supplemental Materials. (**B**) The data shows network interactions in the KEGG signaling pathways. In particular, focal adhesion and the neuroactive ligand–receptor interactions interact largely the others. (**C**) Analysis of gene pathways with high variability for up- and down-regulation of gene expressions.

Rotenone is recognized for its neurotoxic effects in animal studies. In this study, we assessed the impact of rotenone exposure on the expression of genes associated with neurodevelopment and synaptic transmission. RNA sequencing (RNA-seq) analysis revealed that rotenone exposure may suppress ligand–receptor interactions that are important for neurodevelopment (Figure 4A–C). Heatmap analysis of gene expression differences between control and rotenone-treated samples revealed significant alterations in the expression of hundreds of genes. Comprehensive details of upregulated and downregulated genes in control and rotenone-treated samples are provided in a table within the Supplemental Materials. Network interaction analysis within KEGG signaling pathways emphasized substantial involvement in focal adhesion and neuroactive ligand–receptor interaction pathways. This analysis underscores gene pathways exhibiting substantial variability in both upregulated and downregulated gene expression.

### 2.4. Rotenone and Chlorpyrifos Cause Neurodevelopmental Inhibition

**Figure 5:**
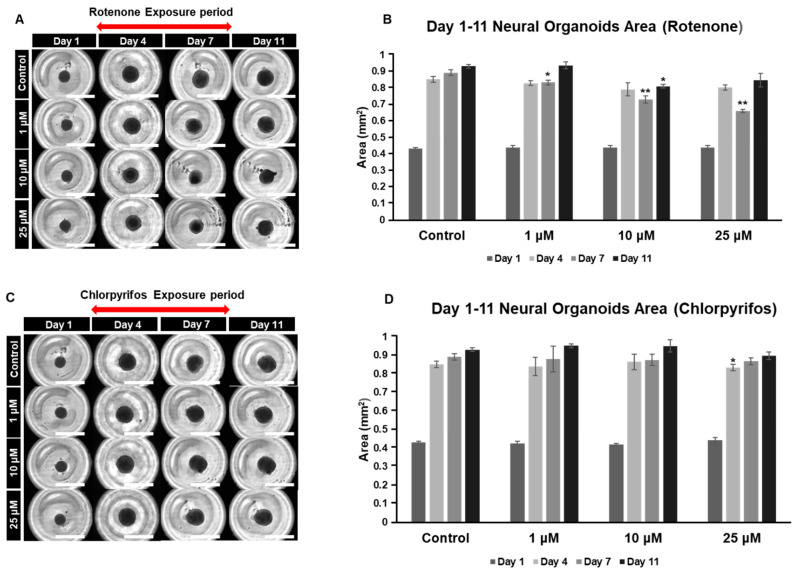
Typical morphological changes in forebrain organoids induced by the pesticides rotenone and chlorpyrifos. (**A**,**B**) Rotenone. (**C**,**D**) Chlorpyrifos. Morphological changes and respective size changes from day 1 to day 11 of incubation. ((**A**,**C**) 4× objective; Scale bar, 2.0 mm). Experiments were performed independently in triplicate or quadruplicate. Asterisks indicate significant differences between the exposed and control groups *p* < 0.05 = *; *p* < 0.01 = **. N = 4.

Chlorpyrifos is an organophosphate pesticide known for its use as a termiticide. Chlorpyrifos, like rotenone, is considered likely to adversely affect the developing nervous system. To investigate the dose-dependent effects of these chemicals on the nervous system, our organoid model was used to analyze morphological changes during the early culture period (days 0 to 11) and the late culture period (days 17 to 29) (Figure 5A–D). The organoids gradually increased in area as the culture period progressed, reaching their maximum size on day 11. Exposure to rotenone resulted in a significant reduction in area compared to the control on day 7. However, by day 11, the size of the organoids, except for those in the 10 μM group, returned to levels similar to the control (Figure 5A,B). In the case of chlorpyrifos, a significant reduction was observed in the 25 μM group on day 4 compared to the control, but no significant differences were found in the other groups (Figure 5C,D).

**Figure 6:**
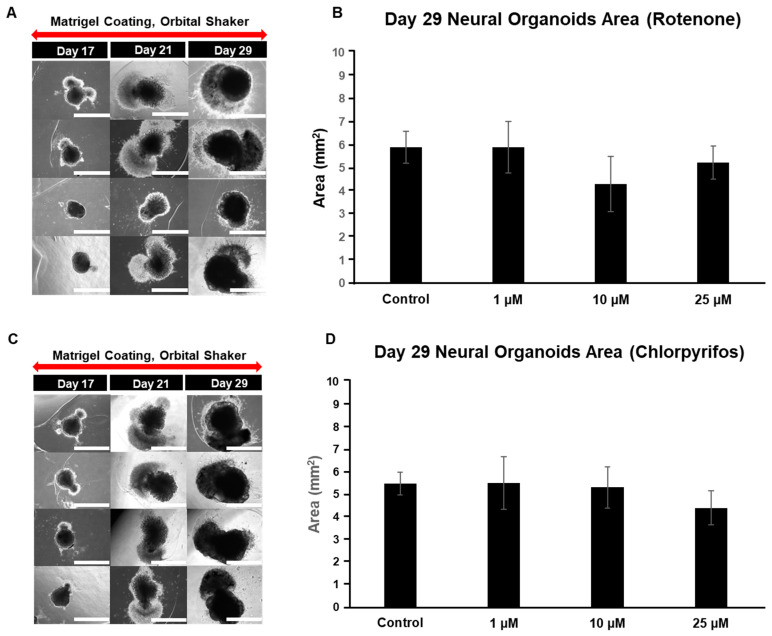
Typical morphological changes in 29-day pre-brain organoids in culture induced by the pesticides rotenone and chlorpyrifos. (**A**,**B**) Rotenone. (**C**,**D**) Chlorpyrifos. Morphological changes and respective size changes on day 29 of incubation. ((**A**,**C**); 4× objective; Scale bar, 2.0 mm). Experiments were performed independently in triplicate or quadruplicate. N = 4.

**Figure 7:**
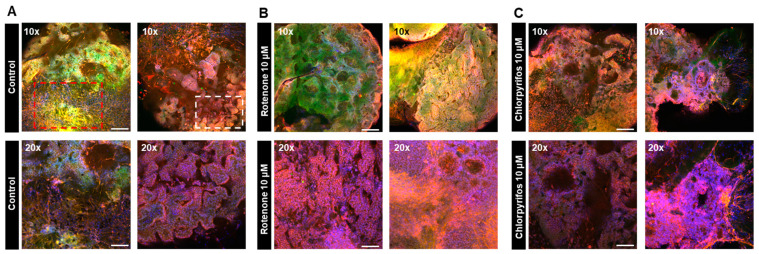
Immunofluorescence images of forebrain-type organoids at 29 days in culture to visualize the effects of chemical substances on neural cell development. (**A**) Internal structure of forebrain-type organoids in the control group, captured in two fields of view (10× and 20× objective). The red boxes indicate nerve dense foci, while the white boxes indicate winding structures. (**B**) Internal structure of organoids exposed to 10 μM rotenone. (**C**) Internal structure of organoids exposed to 10 μM chlorpyrifos. Cell nuclei (blue), neurons (green), and glial cells (red) are shown (*n* = 3; 10× objective; scale bar, 200 μm and 20× objective; scale bar, 100 μm).

**Figure 8:**
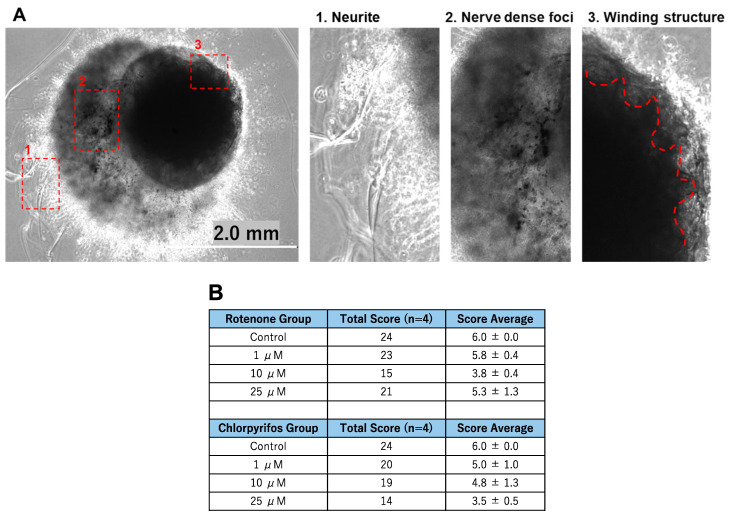
The morphological characteristics of the forebrain organoids identified in Figure 7 were evaluated. (**A**) 1. Neurites were scored as 1 point; 2. nerve dense foci as 2 points; and 3. The red dotted lines indicate three points of the winding structures (scale bar, 2.0 mm). (**B**) Morphological evaluation scores (*n* = 4).

On day 17 of neural organoid differentiation, morphological changes were dramatically observed between the control and pesticides-exposed samples after the Matrigel encapsulation (Figure 6A,C). However, similar changes were not evident in the 10 μM and 25 μM rotenone-exposed samples (Figure 6A,C). On the final day of culture (day 29), it was shown that rotenone had the greatest effect at 10 μM, while chlorpyrifos had the greatest effect at 25 μM (Figure 6B,D). Furthermore, morphological changes associated with neural progenitor cell development were observed at all concentrations of chlorpyrifos, except for 25 μM. Fluorescence immunostaining after 29 days of differentiation revealed that the neural networks (red boxes) and wrinkle-like layered structures (white boxes), which were observed in the control group, were not fully developed in the pesticide-exposed groups (Figure 7). Morphological evaluation scoring revealed the lowest scores for exposure to 10 µM rotenone and 25 µM chlorpyrifos (Figure 8).

**Figure 9:**
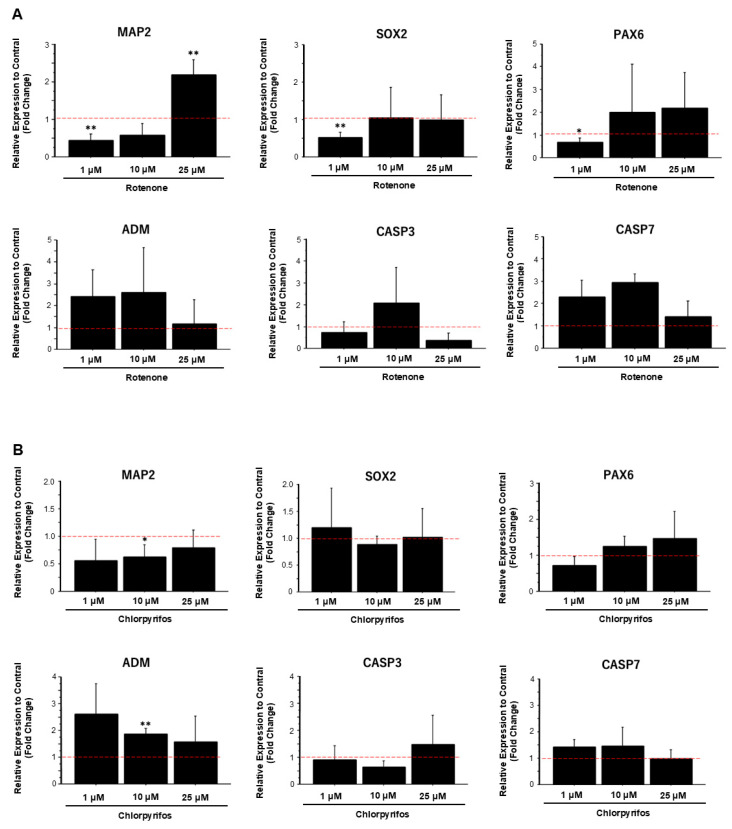
Gene expression analysis of forebrain organoids exposed to rotenone and chlorpyrifos. (**A**) Changes in the expression levels of neuronal marker genes (MAP2, SOX2, PAX6) and apoptosis-related marker genes (ADM, CASP3, CASP7) following exposure to various concentrations of rotenone. (**B**) Changes in the expression levels of neuronal and apoptosis marker genes following exposure to chlorpyrifos. Results are presented as the mean ± SD (*n* = 3). Asterisks indicate *p*-values based on multiple comparisons via two-way analysis of variance *p* < 0.05 = *; *p* < 0.01 = **. The red dotted lines indicate the control value.

To confirm morphological changes, compared with control samples, the expression levels of neural marker genes significantly decreased in samples exposed to low concentrations of rotenone and increased in samples exposed to high concentrations of rotenone (Figure 9A). However, the expression levels of apoptosis-related marker genes did not show any significant change at any concentration. Similarly, exposure to low concentrations of chlorpyrifos reduced the expression levels of neuronal markers genes compared with the control. With increasing chlorpyrifos concentration, the expression levels of the apoptosis-related marker genes adrenomedullin (ADM) and caspase 7 (CASP7) decreased, whereas the expression level of caspase-3 (CASP3) increased (Figure 9B).

## 3. Discussion

Organoid models, unlike two-dimensional neuronal models, are multicellular and functional and closely mimic human brain structures, making them a promising tool for studying toxicity, such as developmental neurotoxicity. Many previous organoid models have been designed to mimic human brain tissue or study diseases and drug screening, making them unsuitable for DNT evaluations due to high costs and the need for cell uniformity in multiple samples [4,21,22]. Therefore, this study attempted to construct neural organoids using human iPSCs as a new method to evaluate the effects of environmental chemicals on the developing nervous system. Moreover, we performed DNT using the pesticides rotenone and chlorpyrifos.

Early exposure to bFGF is known to result in a wrinkle-like appearance of neural organoids [8]. In the current study, on day 24 of differentiation, the formation of neurites from inside the organoids was observed via fluorescence immunostaining (Figure 1). This suggests that bFGF exposure influences early neurogenic processes. Furthermore, on day 50 of differentiation, the bFGF-exposed group formed folded structures with a wrinkled appearance similar to that of the human brain, suggesting that early exposure to bFGF contributes to the structural maturation of neural organoids (Figure 6). These results are considered important factors in the formation of complex tissue structures.

Previous studies on neurotoxicity and neurodevelopmental toxicity have primarily examined the forebrain and central cerebrum, particularly the hippocampus, because these brain regions are predominantly affected by several pesticides and hazardous chemicals [23,24,25]. To develop a more relevant model for safety assessments, we performed differentiation of forebrain-type brain organoids, which offer a more suitable alternative to general neural organoids. Fluorescence immunostaining on day 50 showed strong expression of cytoskeletal markers, indicating well-developed microtubules and microfilaments, which are critical for structural maturation. Although scattered neurons were observed, they lacked projection extension, suggesting functional immaturity (Figure 3). Rotenone is a plant-derived pesticide that inhibits mitochondrial complex I [26]. Despite its short environmental half-life and limited bioavailability, transient exposure to rotenone may impact human health [27,28].

RNA-seq analysis was performed to understand the rotenone-induced changes in gene expression levels within neural organoids. Seventy-eight genes related to ligand–receptor interactions involved in neurodevelopment were significantly altered and showed suppressive changes in expression levels. Hence, rotenone-induced neurotoxicity may affect signaling pathways that are crucial for neurodevelopment.

Rotenone has been shown to induce progressive neurodegeneration not only in dopaminergic neurons but also in nondopaminergic neurons and other brain cell types, such as astrocytes, depending on its concentration [29,30,31]. In animal studies, rotenone caused mitochondrial dysfunction in nondopaminergic central cells and peripheral cells outside the central nervous system [32,33]. In the current study, we selected a concentration range based on previously reported in vitro experiments [34]. On day 17 of neural organoid differentiation, following exposure to 1 μM rotenone, significant morphological changes were observed compared with the control, whereas apoptosis was observed after exposure to 10 and 25 μM rotenone (Figure 5).

Chlorpyrifos is an organophosphate pesticide known for its use as a termiticide. Previous studies, including in vitro and animal experiments [35], have shown that developmental exposure to chlorpyrifos, even at low concentrations, can negatively affect brain maturation. It has been demonstrated that chlorpyrifos can target pathways involved in the etiology of autism spectrum disorder and potentially induce various neurobehavioral disorders as well as structural abnormalities such as cortical thinning [36].

In this study, morphological changes associated with the development of neural progenitor cells were observed after all treatments, except for the 25 μM chlorpyrifos treatment. Fluorescence immunostaining on day 29 of differentiation revealed that the neural networks and groove-like layer structures, which were observed in the control group, were not fully developed in the pesticide-exposed groups (Figure 7). Additionally, morphological evaluation showed that exposure to 10 μM rotenone and 25 μM chlorpyrifos yielded the lowest scores (Figure 7). In contrast, in the assessment of apoptosis-related markers, compared with the control, the expression of ADM—a critical factor that regulates the regeneration and fate of neural stem cells—was significantly suppressed by 25 μM rotenone and 10 μM chlorpyrifos. The gene expression of CASP7, which receives upstream apoptotic signaling and triggers apoptosis by degrading other intracellular proteins, was enhanced by exposure to 1 μM rotenone and 10 μM rotenone, but not by exposure to higher concentrations. Chlorpyrifos exposure did not have a significant impact. These results suggest that rotenone affects both ADM, which is involved in the regulation of neural stem cells, and CASP7, an effector of apoptotic signaling, whereas chlorpyrifos primarily affects ADM, inhibiting neural development (Figure 8). Furthermore, the mechanisms by which rotenone exerts these effects may vary between low and high concentrations. Notably, when exposed to high pesticide concentrations, despite the smaller size of EBs at early developmental stages, neural outgrowth was significantly larger than that of the control, indicating the potential to maintain self-homeostasis. This suggests that the forebrain organoids developed in this study possess self-regulating mechanisms necessary to adapt to changes in the external environment and sustain survival. The pathophysiological model of Kleefstra syndrome (KS) developed by Balogh et al. utilizes a 3D cellular system, demonstrating reduced cell proliferation, alterations in neurotransmitter signaling, and increased sensitivity to oxidative stress. This model highlights crucial molecular pathways that may serve as therapeutic targets [37].

In contrast, our 3D neural model possesses structures and functions that more closely resemble actual neural tissue, making it highly sensitive to chemical and environmental stressors. This sensitivity allows for earlier and more precise detection of the effects of chemicals on neurodevelopment compared to conventional models. In this study, our model also demonstrated a comparable level of sensitivity to rotenone as seen in previous studies, further validating the utility of our 3D organoid model.

## 4. Materials and Methods

### 4.1. Chemicals

A stock solution of recombinant human bFGF (Wako, Osaka, Japan) was prepared at a concentration of 5 μg/mL in dimethyl sulfoxide (DMSO; Nacalai, Kyoto, Japan). Stock solutions of rotenone (TCI) and chlorpyrifos (Wako, Osaka, Japan) were also prepared in DMSO.

### 4.2. Maintenance Culture of Human iPSCs

We used the human iPSC line HPS0003 derived from human umbilical cord cells, which were obtained from RIKEN. The cells were maintained in serum-free maintenance medium (mTeSR Plus) on 35-mm^2^ dishes that were precoated with Matrigel as the cell basement membrane. On the first day of cell thawing and passaging, the apoptosis inhibitor Y-27632 (Wako, Osaka, Japan) was added at a final concentration of 10 μM. The culture medium was replaced the day after seeding and every other day subsequently. Culture was performed at 37 °C under 5% CO_2_.

### 4.3. Differentiation of Neural Organoids

HPS0003 cells that reached 80% confluency were detached using the cell dissociation enzyme Accutase and then counted. The cells were seeded at a density of 9000 cells per well in a V-bottomed 96-well plate. The cells were cultured in EB formation medium (mTeSR Plus + 10% FBS) supplemented with Y-27632 at a final concentration of 50 μM for 24 h; then, the medium was replaced with EB formation medium without Y-27632 and cultured until day 4. After EB formation, the cells were cultured until day 15 in neural induction medium (STEMCELL Technologies, Vancouver, BC, Canada) supplemented with a TGF-β/SMAD inhibitor. On day 15 of differentiation, the medium was replaced with forebrain neural differentiation medium (STEMCELL Technologies, Vancouver, BC, Canada), and each EB was embedded in 15 μL of Matrigel. The EBs were then transferred to a low-adhesion 6-well plate, with five EBs per well, and incubated undisturbed for 24 h. On day 16, the culture was maintained in an orbital shaker (65 rpm, 25 mm). On day 22, the medium was replaced with forebrain neural maturation medium (STEMCELL Technologies, Vancouver, BC, Canada) and maintained until day 29. The culture conditions were 5% CO_2_ at 37 °C; the shaking parameters were 30 rpm and 20 mm.

### 4.4. RNA Sequencing Analysis

RNA-seq raw data was processed using Salmon version (1.10.3) on macOS (Apple Inc., Cupertino, CA, USA). The raw sequencing reads (FASTQ files) were aligned to the reference transcriptome using Salmon’s quasi-mapping algorithm, which provides a robust and efficient method for transcript quantification. Subsequently, RStudio version (2024.04.2+764) was used for further data analysis. For visualization and pathway analysis, the iDEP.96 web application was utilized.

### 4.5. DNT of Rotenone and Chlorpyrifos

During the neural induction phase of neural organoid differentiation, the pesticides rotenone (1, 10, and 25 μM) and chlorpyrifos (1, 10, and 25 μM) were applied for 72 h between days 4 and 7. After exposure, the medium was removed, and the organoids were fixed at 4 °C overnight in a 4% paraformaldehyde solution. After fixation, paraformaldehyde was removed, and the organoids were washed twice with phosphate-buffered saline (PBS). The organoids were mounted in a microchamber using the tissue clearing agent RapiClear 1.52 and then imaged. Images were acquired using an Olympus confocal microscope, and the internal structure of the organoids were evaluated using the image analysis software FV10-ASW Version 04.02.02.09.

### 4.6. RNA Extraction

Total RNA from forebrain neural organoids was collected on day 29 of differentiation. Additionally, organoids exposed to 1, 10, and 25 μM rotenone and chlorpyrifos for 72 h were also collected. Total RNA was extracted using RNeasy Mini Kit (Qiagen, Hilden, Germany). The quantity and quality of the RNA were measured using a Nano-Drop Lite (Thermo Fisher Scientific, Waltham, MA, USA). The extracted RNA was reverse-transcribed using PrimeScript RT Reagent Kit (Takara Bio, Shiga, Japan). cDNA synthesis was performed using a thermal cycler (GeneAmp PCR System 9700). The obtained cDNA was subjected to real-time polymerase chain reaction using the Fast Real-Time System machine (Applied Biosystems, Foster City, CA, USA).

### 4.7. Gene Expression Analysis

Gene expression was analyzed using various markers: MAP2 as a marker for mature neurons; SOX2 and PAX6 as markers for neural progenitor cells; and the apoptosis-related markers ADM, which exhibits antiapoptotic effects, and CASP3 and CASP7, which are associated with the degradation of cytoplasmic and nuclear proteins. Gene expression was quantified using the SYBR Green gene expression assay (Thermo Fisher Scientific, Waltham, MA, USA) via StepOne Plus (Applied Biosystems, Foster City, CA, USA). The variation in gene expression levels was calculated using the 2^−ΔΔCt^ method (Livak and Schmittgen, 2001) to determine relative gene expression.

### 4.8. Fluorescence Immunostaining

Forebrain neural organoids were collected on day 29 of differentiation. The collected organoids were fixed overnight in 4% paraformaldehyde and then washed twice with PBS. After fixation, the organoids were incubated overnight in a blocking solution (5% bovine serum albumin [BSA] in PBS/1% Triton-X100). Next, the organoids were mixed with PBS containing 0.1% Tween 20 and incubated at room temperature for 30 min. After washing the organoids twice with PBS, primary antibodies diluted in 1% BSA/0.1% Triton-X100 in PBS were added and incubated at 4 °C for 48 h under gentle agitation. The organoids were then washed twice with PBS and incubated with secondary antibodies in 1% BSA/0.1% Triton-X100 in PBS at 4 °C for 48 h. Before mounting the organoids in microchambers for confocal observation using RapiClear 1.52, they were stained for 1 h under dark conditions with Hoechst 33,342 (Wako; 1:1000 in PBS), Neuronal Marker (MILLIPORE; 1:100 in PBS), GFAP (PGI; 1:1000) and Phalloidin Rhodamine (Sigma; 1:60). Forebrain neural organoids at day 50 of culture were similarly stained, and images were acquired using Nikon AX/AX R super-resolution confocal laser microscopy system.

## 5. Conclusions

The neural organoid model developed in this study demonstrates the potential to assess the effects of pesticides on the developing brain and represents a reproducible, innovative tool for DNT studies. Due to its multicellular architecture, this model provides a multidimensional assessment of chemical substances, enabling the exploration of cellular interactions and mechanisms in a manner that closely reflects physiological conditions. Furthermore, this organoid model accurately recapitulates aspects of human neurodevelopment, which is anticipated to advance the depth and precision of future research in this field.

## Data Availability

The NGS data are publicly available on the GEO website under accession number GSE282273. Differential gene expression data are provided in the Supplementary Materials.

## Supplementary Materials

The following supporting information can be downloaded at: https://www.mdpi.com/article/10.3390/ijms252312523/s1.
